# Sub‐cellular location of FtsH proteases in the cyanobacterium *S* *ynechocystis* sp. PCC 6803 suggests localised PSII repair zones in the thylakoid membranes

**DOI:** 10.1111/mmi.12940

**Published:** 2015-02-11

**Authors:** Joanna Sacharz, Samantha J. Bryan, Jianfeng Yu, Nigel J. Burroughs, Edward M. Spence, Peter J. Nixon, Conrad W. Mullineaux

**Affiliations:** ^1^ School of Biological and Chemical SciencesQueen Mary University of LondonMile End RoadLondonE1 4NSUK; ^2^ Department of Life SciencesImperial College LondonSouth Kensington CampusLondonSW7 2AZUK; ^3^ Systems Biology CentreCoventry HouseUniversity of WarwickCoventryCV4 7ALUK; ^4^ Institute of Pharmaceutical ScienceKing's College LondonLondonSE1 9NHUK

## Abstract

In cyanobacteria and chloroplasts, exposure to HL damages the photosynthetic apparatus, especially the D1 subunit of Photosystem II. To avoid chronic photoinhibition, a PSII repair cycle operates to replace damaged PSII subunits with newly synthesised versions. To determine the sub‐cellular location of this process, we examined the localisation of FtsH metalloproteases, some of which are directly involved in degrading damaged D1. We generated transformants of the cyanobacterium *S* *ynechocystis* sp. PCC6803 expressing GFP‐tagged versions of its four FtsH proteases. The ftsH2–gfp strain was functional for PSII repair under our conditions. Confocal microscopy shows that FtsH1 is mainly in the cytoplasmic membrane, while the remaining FtsH proteins are in patches either in the thylakoid or at the interface between the thylakoid and cytoplasmic membranes. HL exposure which increases the activity of the Photosystem II repair cycle led to no detectable changes in FtsH distribution, with the FtsH2 protease involved in D1 degradation retaining its patchy distribution in the thylakoid membrane. We discuss the possibility that the FtsH2–GFP patches represent Photosystem II ‘repair zones’ within the thylakoid membranes, and the possible advantages of such functionally specialised membrane zones. Anti‐GFP affinity pull‐downs provide the first indication of the composition of the putative repair zones.

## Introduction

The photosynthetic apparatus of cyanobacteria, including the Photosystem I (PSI) and Photosystem II (PSII) reaction centres, is housed in the thylakoid membranes, a complex internal membrane system (Gantt, 1994). Thylakoid biogenesis is a poorly understood process, with many unanswered questions concerning the sub‐cellular location of the complex sequence of steps required to assemble the reaction centre protein complexes and their co‐factors (Mullineaux, 1999). Early biochemical evidence suggested that some steps in the process may occur in the cytoplasmic membrane rather than the thylakoids (Smith and Howe, 1993; Zak *et al*., 2001), while current models suggest that reaction centres are assembled in centres in close proximity to both the cytoplasmic and thylakoid membranes (Stengel *et al*., 2012). Since the mobility of membrane‐integral proteins, and especially PSII, in the thylakoid membrane is very restricted (Mullineaux *et al*., 1997; Sarcina and Mullineaux, 2004), the question of how reaction centres can migrate from assembly sites to take their place in the mature membrane is not trivial. A related problem concerns the site(s) of repair of photodamaged reaction centres. Reaction centre subunits (and especially the core D1 subunit of PSII) are continually photodamaged and must be removed from the complex, degraded and replaced with newly synthesised protein (Ohad *et al*., 1984; Edelman and Mattoo, 2008; Nixon *et al*., 2010). Photoinhibition of photosynthesis results if reaction centre repair does not keep pace with photodamage (Aro *et al*., 1993). Biochemical and mutagenesis studies have shed considerable light on the Photosystem II repair cycle in cyanobacteria, with the identification of repair factors including proteases required to degrade damaged D1 subunits (Nixon *et al*., 2010). However, the location of the repair cycle remains uncertain, with some models suggesting that steps in the process take place in the cytoplasmic membrane (Smith and Howe, 1993; Zak *et al*., 2001; Nowaczyk *et al*., 2006).

The FtsH family of zinc metalloproteases are considered to play a key role in D1 degradation in both chloroplasts and cyanobacteria (reviewed by Adam and Clarke, 2002; Nixon *et al*., 2005). FtsH proteases are membrane‐integral proteins belonging to the AAA+ (ATPases associated with diverse cellular activities) superfamily (Ito and Akiyama, 2005). FtsH (*HlfB*) was originally identified in *Escherichia coli* as one of five proteases representative of the AAA+ superfamily, and the only one essential for viability (Ogura *et al*., 1999). FtsH in *E. coli* is found in the cytoplasmic membrane (Tomoyasu *et al*., 1995) and maintains quality control through degradation of misfolded or damaged membrane proteins (Akiyama, 2009). FtsH proteases are evolutionarily conserved in eubacteria, mitochondria and chloroplasts (Langklotz *et al*., 2012). While *E. coli* has only a single *ftsH* gene, cyanobacterial genomes have multiple *ftsH* homologues. The model cyanobacterium *Synechocystis* sp. PCC 6803 has four FtsH proteases: FtsH1 (Slr1390), FtsH2 (Slr0228), FtsH3 (Slr1604) and FtsH4 (Sll1463). Insertional null mutants in *slr1390* and *slr1604* failed to segregate, indicating essential but unknown functions, while a null mutant of *sll1463* segregated but had no readily apparent phenotypic defect (Mann *et al*., 2000). However, Δ*slr0228* had a light‐sensitive phenotype, with a strongly disabled Photosystem II repair cycle (Silva *et al*., 2003). Subsequent studies confirmed a specific role for FtsH2 in D1 turnover, the Photosystem II repair cycle and protein quality control in the thylakoid membrane (Komenda *et al*., 2006; 2010). Membrane fractionation studies suggest that FtsH2 is found in the thylakoids (Komenda *et al*., 2006; Pisareva *et al*., 2007), as is FtsH4 (Pisareva *et al*., 2007). Membrane fractionation also suggests that FtsH1 and FtsH3 are found in the plasma membrane (Pisareva *et al*., 2007), but it should be noted that membrane fractionation may not always give a complete or reliable picture of sub‐cellular localisation, especially if membrane systems are laterally heterogeneous. The recent characterisation of an intermediate‐density sub‐fraction apparently devoted to reaction centre biogenesis (Stengel *et al*., 2012) is an indication of extra complexity in the system. It is likely that the FtsH subunits are present in hexameric complexes in the membrane, with recent data identifying the presence of FtsH2/FtsH3, FtsH1/FtsH3 and FtsH4 complexes (Boehm *et al*., 2012). The FtsH1/FtsH3 complex was recently shown to be important in acclimation to iron deficiency by controlling the level of the Fur repressor (Krynická *et al*., 2014).

To shed more light on the roles and interactions of the FtsH proteins and the sub‐cellular location of the PSII repair cycle, we set out to visualise all four FtsH proteins in *Synechocystis* cells *in vivo* by tagging each protease with enhanced Green Fluorescent Protein (eGFP). We show that all the FtsH proteins are concentrated in distinct patches in the thylakoid or cytoplasmic membranes: in the case of FtsH2, these could correspond to PSII repair zones in the thylakoids. Anti‐GFP affinity pull‐downs from the *ftsH2–gfp* strain isolate a distinct thylakoid membrane sub‐fraction, giving a first indication of the protein content of these membrane zones.

## Results

### GFP tagging of FtsH proteins

**Figure 1:**
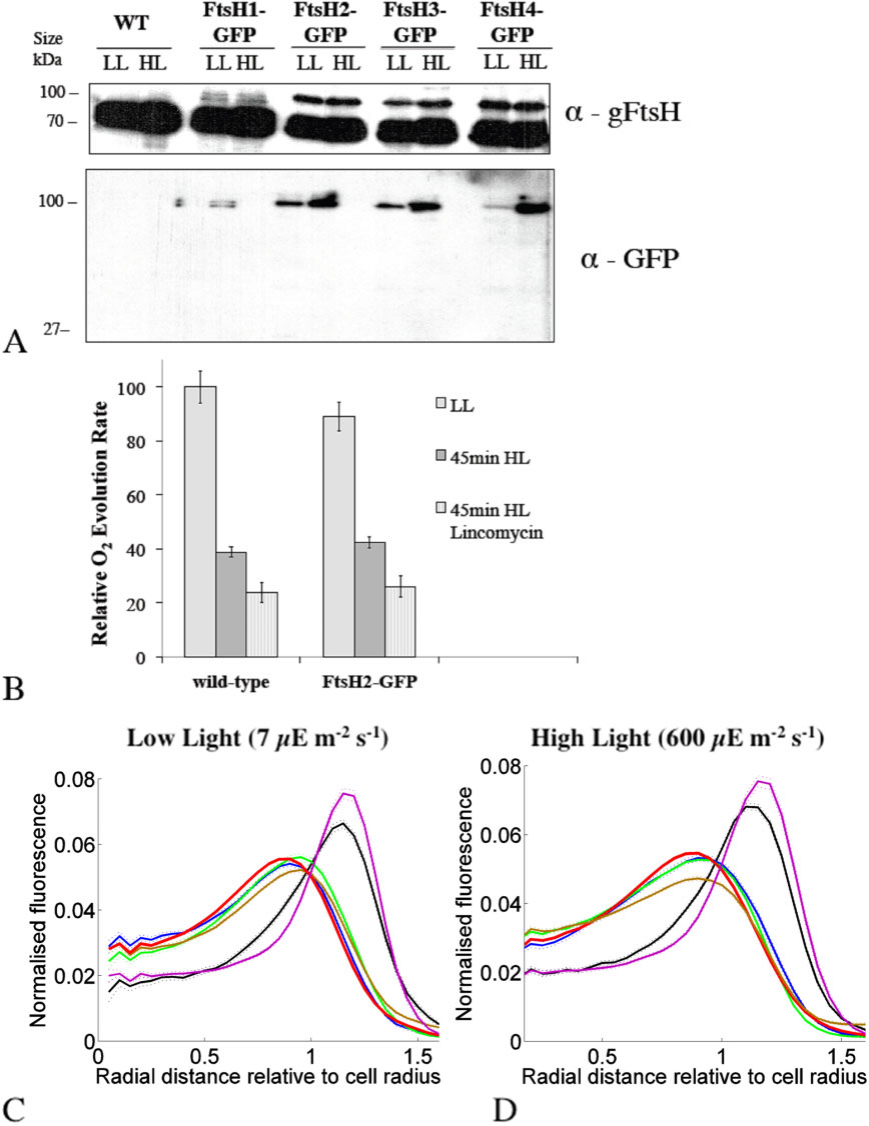
Characterisation of *S* *ynechocystis* ftsH–gfp mutants. A. Immunoblot analysis, lanes loaded with proteins of crude membranes isolated from the four ftsH–gfp mutants (5 μg chlorophyll per lane), separated by SDS–PAGE 10% (w/v) polyacrylamide gels and blotted with anti‐GFP and global anti‐FtsH antibody, recognising all four FtsH proteins. B. Photoinhibition and the PSII repair cycle in wild‐type and ftsH2–gfp cells. Relative oxygen evolution rates (light saturated and in the presence of PSII electron acceptor) for cells grown in LL and exposed to high light for 45 min ± lincomycin to inhibit protein synthesis and therefore the PSII repair cycle. O_2_ evolution rates are shown relative to the maximum rate observed in LL cells. Means from three biological replicates, with standard deviations. Differences between the three conditions are all significant (Student's *t‐*test, *P* < 0.05). C and D. Radial distributions of fluorescence under low light (C) and high light (D) for chlorophyll (red line), FtsH1–GFP (black line), FtsH2–GFP (blue line), FtsH3‐GFP (brown line), FtsH4–GFP (green line) and periplasmic FutA1–GFP (magenta line). Data averaged from a minimum of 443 cells and normalised to total fluorescence from each species. A standardised cell radius is defined by chlorophyll fluorescence.

To investigate the subcellular localisation of each of the FtsH proteases (FtsH1–4) in cells of *Synechocystis* sp. PCC6803 *in vivo*, we generated transformants expressing in‐frame C‐terminal eGFP fusions to each protein. To ensure that expression of the FtsH proteins was in context and physiologically relevant, the gene fusions were introduced into the native chromosomal loci, replacing the wild‐type genes but retaining the native promoter (Fig. S1A). Segregation of all the *ftsH–gfp* strains was complete (i.e. the wild‐type loci were replaced by the mutant loci in all of the multiple copies of the chromosome) (Fig. S1B). Insertional null mutants in *ftsH1* and *ftsH3* do not segregate (Mann *et al*., 2000), indicating an indispensable function for these genes. Therefore, the fact that the *ftsH1–gfp* and *ftsH3–gfp* strains are able to segregate (Fig. S1B) indicates that FtsH1–GFP and FtsH3–GFP retain function. Given that Δ*ftsH2* shows a light‐sensitive phenotype, with the PSII repair cycle strongly impaired (Silva *et al*., 2003), we compared susceptibility with photoinhibition in the wild‐type and *ftsH2–gfp* to check for the functionality of FtsH2–GFP. Under photoinhibitory conditions, there was no significant difference in the maintenance of PSII oxygen‐evolving activity between the wild‐type and *ftsH2–gfp* (Fig. 1B). This is in marked contrast to the previously characterised *ftsH2* null mutants (Silva *et al*., 2003), and it demonstrates that the GFP tag does not block the function of FtsH2 in the PSII repair cycle. Likewise, fusion of a GST tag to the C‐terminus of FtsH2 has also been shown not to affect FtsH2 function (Boehm *et al*., 2012).

Immunoblots with a global anti‐FtsH antibody (Fig. 1A) reveal the presence of a ∼ 70 kDa band corresponding to the unmodified FtsHs: note that each tagged strain will retain the wild‐type versions of the other three FtsHs. A higher molecular‐weight band with size corresponding to the expected size of FtsH–GFP (∼ 100 kDa) is seen in all four *ftsH–gfp* strains. This higher molecular‐weight band is absent from the wild‐type, as expected (Fig. 1A). The blot for *ftsH1–gfp* shows doublet bands around 100 kDa, which might be a result of protein cleavage in the transmembrane region during sample preparation (Fig. 1A). Immunoblots with anti‐GFP antibody show that all detectable GFP in the cells is linked to proteins of the expected size, with no free GFP detected in the thylakoid fraction (Fig. 1A) or in the soluble fraction (not shown). Immunoblots with specific anti‐FtsH antibodies show that each specific FtsH protein is linked to GFP in the appropriate *ftsH–gfp* strain (Fig. S2).

### Localisation of FtsH proteins

Confocal fluorescence microscopy was used to visualise the localisation of the FtsH proteases in cells of *Synechocystis* sp. PCC 6803 grown under low light (LL), or following 1‐hour high‐light (HL) exposure. Excitation was at 488 nm and fluorescence emission was recorded simultaneously for GFP in the green (502–512 nm) and for chlorophyll in the red (670–720 nm). Chlorophyll fluorescence indicates the location of the thylakoid membranes (Mullineaux and Sarcina, 2002). For comparison, control images were recorded for a FutA1–GFP fusion which is localised to the periplasm (Bryan *et al*., 2014). *Synechocystis* cells are approximately spherical. Therefore, to quantify the localisation of GFP fluorescence, cell images were segmented into cytoplasmic, thylakoid and periplasmic/cytoplasmic membrane regions based on the radial distribution of fluorescence relative to a standardised cell radius (Fig. 1C and D). Comparison with the radial distributions of chlorophyll and FutA1–GFP fluorescence (Fig. 1C and D) showed that FtsH1 is present mainly in the plasma membrane. However, FtsH1 shows more fluorescence overlapping the thylakoid region than FutA1–GFP (Fig. 1C and D) suggesting the likelihood of a minor population of FtsH1 in the thylakoids. The radial distribution of FtsH2 corresponded closely to the radial distribution of chlorophyll, suggesting that this protein is exclusively found in the thylakoid membrane (Fig. 1C and D). Radial distributions show that FtsH3 and FtsH4 are mainly found in the thylakoids, but both show fluorescence distributions weighted to the distal side of the chlorophyll fluorescence, suggesting a concentration at the distal edge of the thylakoids and/or a sub‐population in the cytoplasmic membrane (Fig. 1C and D). This would be consistent with an FtsH1/FtsH3 hetero‐oligomer in the cytoplasmic membrane (Krynická *et al*., 2014).

**Figure 2:**
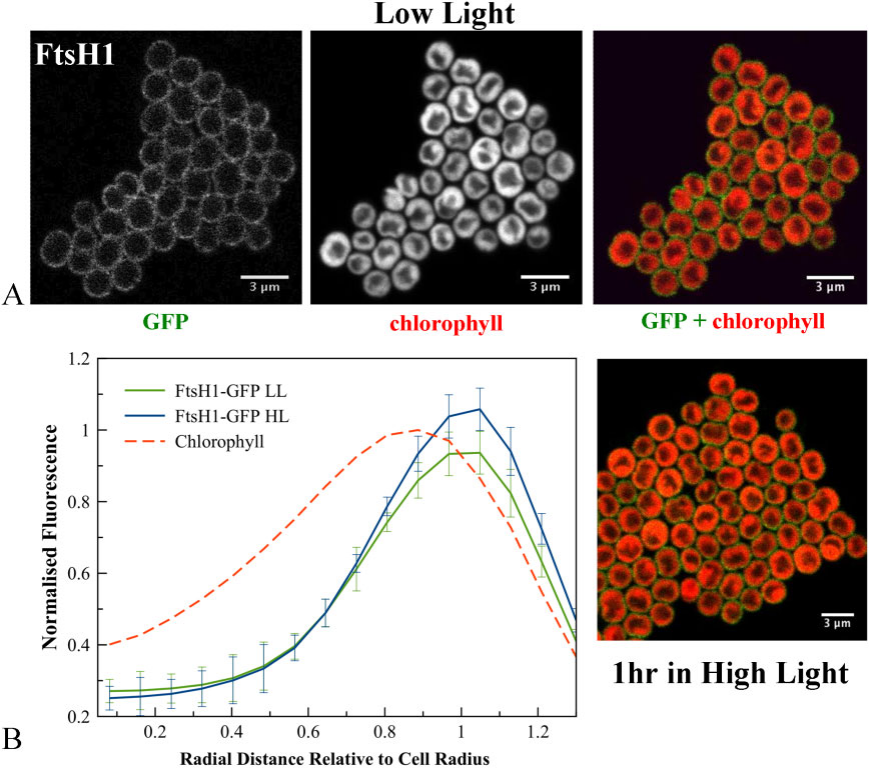
A. Confocal fluorescence images of *S* *ynechocystis* ftsH1–gfp cells, showing GFP fluorescence, chlorophyll fluorescence and merged images (GFP in green, chlorophyll in red) for cells grown in low light or exposed to high light. B. Averaged fluorescence radial distributions (*n* = 30, for three biological replicates), standardised to cell radius and normalised to the maximum chlorophyll peak in LL. Red: chlorophyll fluorescence in LL cells; green: GFP fluorescence in LL; blue: GFP fluorescence in HL. Error bars indicate standard deviations.

**Figure 3:**
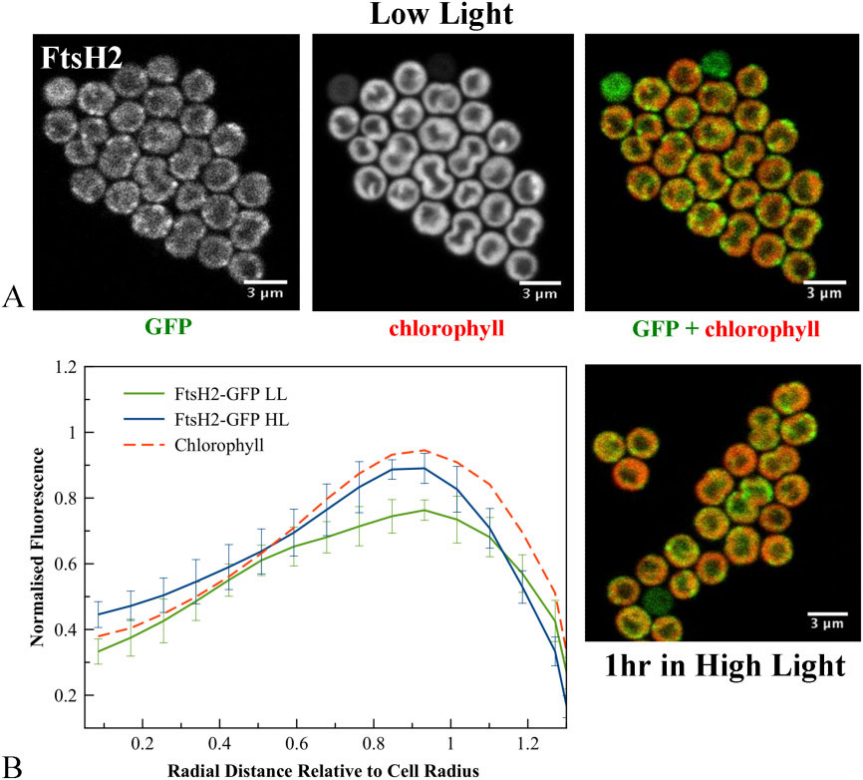
A. Confocal fluorescence images of *S* *ynechocystis* ftsH2–gfp cells, showing GFP fluorescence, chlorophyll fluorescence and merged images (GFP in green, chlorophyll in red) for cells grown in low light or exposed to high light. B. Averaged fluorescence radial distributions (*n* = 30, for three biological replicates), standardised to cell radius and normalised to the maximum chlorophyll peak in LL. Red: chlorophyll fluorescence in LL cells; green: GFP fluorescence in LL; blue: GFP fluorescence in HL. Error bars indicate standard deviations.

**Figure 4:**
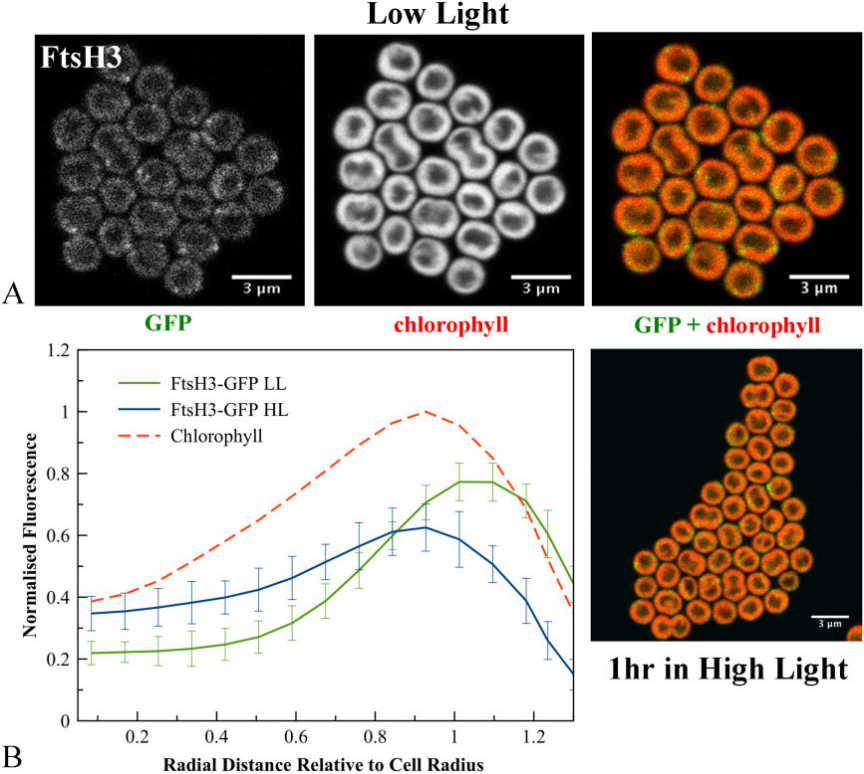
A. Confocal fluorescence images of *S* *ynechocystis* ftsH3–gfp cells, showing GFP fluorescence, chlorophyll fluorescence and merged images (GFP in green, chlorophyll in red) for cells grown in low light or exposed to high light. B. Averaged fluorescence radial distributions (*n* = 30, for three biological replicates), standardised to cell radius and normalised to the maximum chlorophyll peak in LL. Red: chlorophyll fluorescence in LL cells; green: GFP fluorescence in LL; blue: GFP fluorescence in HL. Error bars indicate standard deviations.

**Figure 5:**
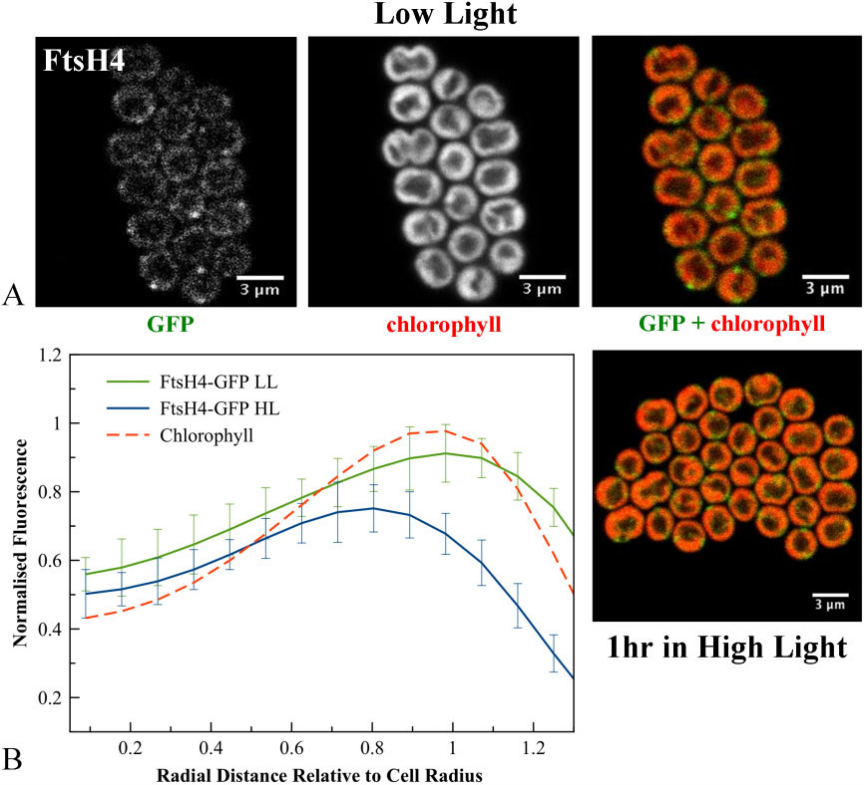
A. Confocal fluorescence images of *S* *ynechocystis* ftsH4–gfp cells, showing GFP fluorescence, chlorophyll fluorescence and merged images (GFP in green, chlorophyll in red) for cells grown in low light or exposed to high light. B. Averaged fluorescence radial distributions (*n* = 30, for three biological replicates), standardised to cell radius and normalised to the maximum chlorophyll peak in LL. Red: chlorophyll fluorescence in LL cells; green: GFP fluorescence in LL; blue: GFP fluorescence in HL. Error bars indicate standard deviations.

Inspection of fluorescence images for individual cells (Figs 2, 3, 4, 5) revealed that none of the FtsH proteins are evenly distributed within the membrane. As compared with chlorophyll fluorescence, which is generally rather uniformly distributed within the thylakoid membrane system (Mullineaux and Sarcina, 2002), FtsH–GFP fluorescence is clearly more patchy. FtsH1 shows a rather patchy distribution within the cytoplasmic membrane (Fig. 2A), while FtsH2 is concentrated in distinct patches in the thylakoid membrane (Fig. 3A). Previous observations at lower resolution also show that the distribution of FtsH2–GFP in the thylakoid membrane is distinct from that of chlorophyll (Komenda *et al*., 2006; Krynická *et al*., 2014). The number of FtsH2 patches per cell varies from about 2 to 5 in the 1 μm optical sections that we observe (Fig. 3A), which will include roughly 60% of the cell volume. Therefore, cells must typically contain about three to eight distinct patches of FtsH2 in their thylakoid membranes. In extreme cases, extended patches enriched in FtsH2 occupy a significant part of the thylakoid (Fig. 3A).

FtsH3 is concentrated in small spots usually at the periphery of the thylakoid membranes. Usually two to three spots were observed per cell, which implies three to five spots of FtsH3 in the complete cell (Fig. 4A). Averaged line profiles for fluorescence confirm that a significant proportion of FtsH3–GFP fluorescence is more distal to the cell centre than chlorophyll fluorescence (Fig. 4B), indicating that some FtsH3–GFP spots are either at the outer periphery of the thylakoids or in the cytoplasmic membrane. FtsH4 shows a similar distribution to FtsH3, with spots mainly at the outer periphery of the thylakoid membranes (Fig. 5A).

We estimated the true dimensions of FtsH–GFP patches in the membrane by correcting for optical spread with the method employed by Rexroth *et al*. (2011). Fluorescence profiles for GFP fluorescence were extracted from the images both in the membrane plane and perpendicular to the membrane. If it assumed that the patches are confined to a single membrane plane, then their dimensions in the profile perpendicular to the membrane are negligible at optical resolution, and this profile approximately reflects the line‐spread function of the microscope. Subtracting the diameter (full‐width at half‐maximum) of the perpendicular profile from the profile in the membrane plane gives a corrected estimate for the diameter of the patch (Rexroth *et al*., 2011). We found that the diameters of the thylakoid membrane patches defined by FtsH2–GFP were in the range from 200 to 600 nm, with a mean diameter of 370 ± 140 nm [standard deviation (S.D., *n* = 50)]. Thylakoid membrane patches defined by FtsH3–GFP were generally slightly smaller, with diameters in the range from 100 to 300 nm and mean diameter 210 ± 80 nm (S.D., *n* = 50).

### The patchy distribution of FtsH proteases is maintained in high light

Because of the well‐established role of FtsH2 in the PSII repair cycle (Silva *et al*., 2003; Komenda *et al*., 2006), it is of interest to establish the localisation of the FtsH proteins under conditions in which the activity of the repair cycle is high. We induced photoinhibition by exposure of cultures grown in LL to HL at 600 μE m^−2^ s^−1^ for 45–60 min. Measurement of PSII oxygen‐evolving activity shows that PSII was significantly photodamaged under these conditions (Fig. 1B). In the presence of lincomycin to inhibit protein synthesis (Dalla Chiesa *et al*., 1997), the loss of PSII activity was significantly higher, indicating that protein synthesis is mitigating the effects of PSII photodamage, and therefore that the PSII repair cycle is active (Silva *et al*., 2003) (Fig. 1B).

**Figure 6:**
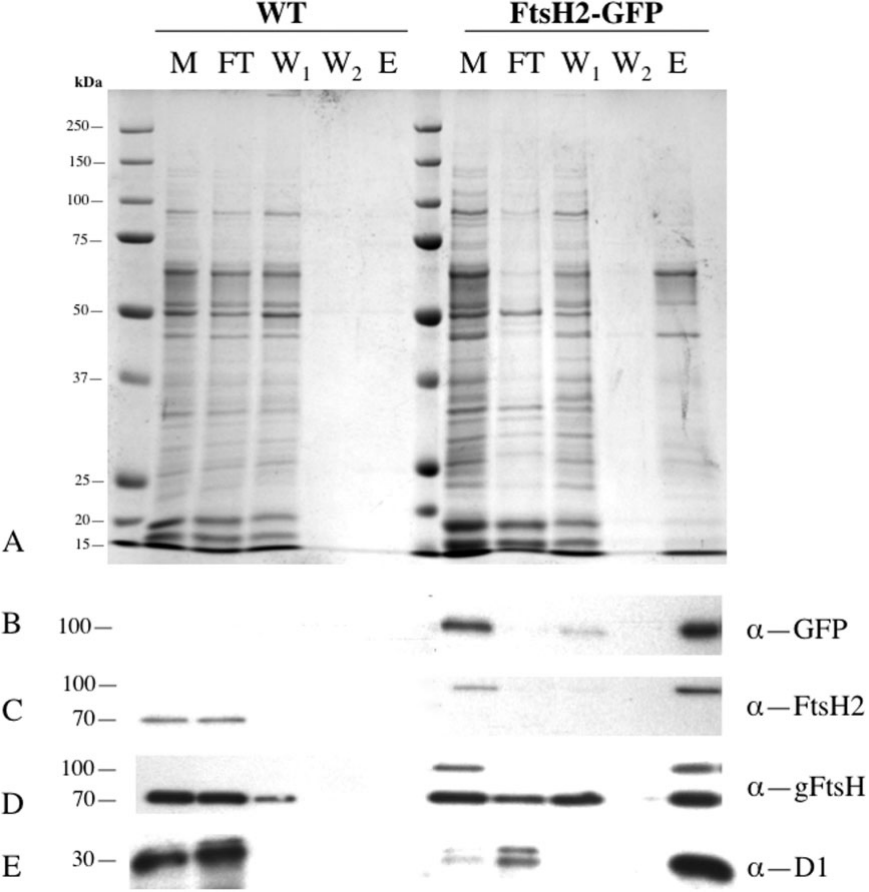
Use of anti‐GFP pull‐downs to isolate a thylakoid membrane fraction from low‐light *S* *ynechocystis* ftsH2–gfp cells. A. Silver‐stained SDS–PAGE gel for membrane fractions from wild‐type and ftsH2–gfp. M: crude membrane preparation (material containing 0.1 nmol chlorophyll loaded per lane); FT: flow‐through (i.e. unbound material) from the column; W_1_, W_2_: first and second column washes; E: final elute of bound material. Each of lanes FT, W_1_, W_2_ and E contains the appropriate fraction from material containing 0.2 nmol chlorophyll loaded onto the column. B–E. Immunoblots with antibodies against GFP (B), FtsH2 (C), FtsH (global) (D) and D1 protein of PSII (E).

The radial distributions of FtsH1 and FtsH2 were not significantly altered by HL treatment, with FtsH2 fluorescence still found in the thylakoid region and FtsH1 mainly in the cytoplasmic membrane (Figs 1D, 2B, 3B). However, both FtsH3 and FtsH4 significantly shifted their distributions inwards from the outer periphery of the thylakoids, with their radial distributions after HL exposure closer to that of chlorophyll (Figs 4B and 5B). Chlorophyll fluorescence did not significantly change in distribution after HL exposure (Fig. S3). HL did not induce any observable differences in the patchy distribution of FtsH1 and FtsH2 within the membranes (Figs 2 and 3). The mean corrected total cell fluorescence for FtsH2–GFP increased by about 40% after HL exposure (Fig. 3), consistent with the immunoblots which also suggest increased FtsH2–GFP content relative to chlorophyll (Fig. 1A) but without significant effect on the radial distribution (Figs 1D and 3B). Despite its increased expression under HL, FtsH2 remained concentrated in thylakoid membrane patches similar to those observed under LL (Figs 3 and 6). To quantify the extent to which FtsH2 is localised in segregated thylakoid membrane patches as opposed to being evenly spread in the thylakoid membranes, we calculated correlation coefficients for co‐localisation of chlorophyll and GFP fluorescence in the two conditions (Fig. S4). We reasoned that segregation of FtsH2–GFP into distinct thylakoid membrane zones would decrease the correlation between GFP and chlorophyll fluorescence, while a more even distribution of FtsH2 in the membrane would have the opposite effect. However, neither Pearson's correlation coefficient nor Manders' overlap coefficient (Manders *et al*., 1993; Zinchuk *et al*., 2007) was significantly affected by HL treatment (Fig. S4B), indicating that the relative distributions of FtsH2–GFP and chlorophyll fluorescence do not change significantly under conditions when the PSII repair cycle is active (Fig. S4B).

### Isolation of membrane fragments associated with FtsH2

**Figure 7:**
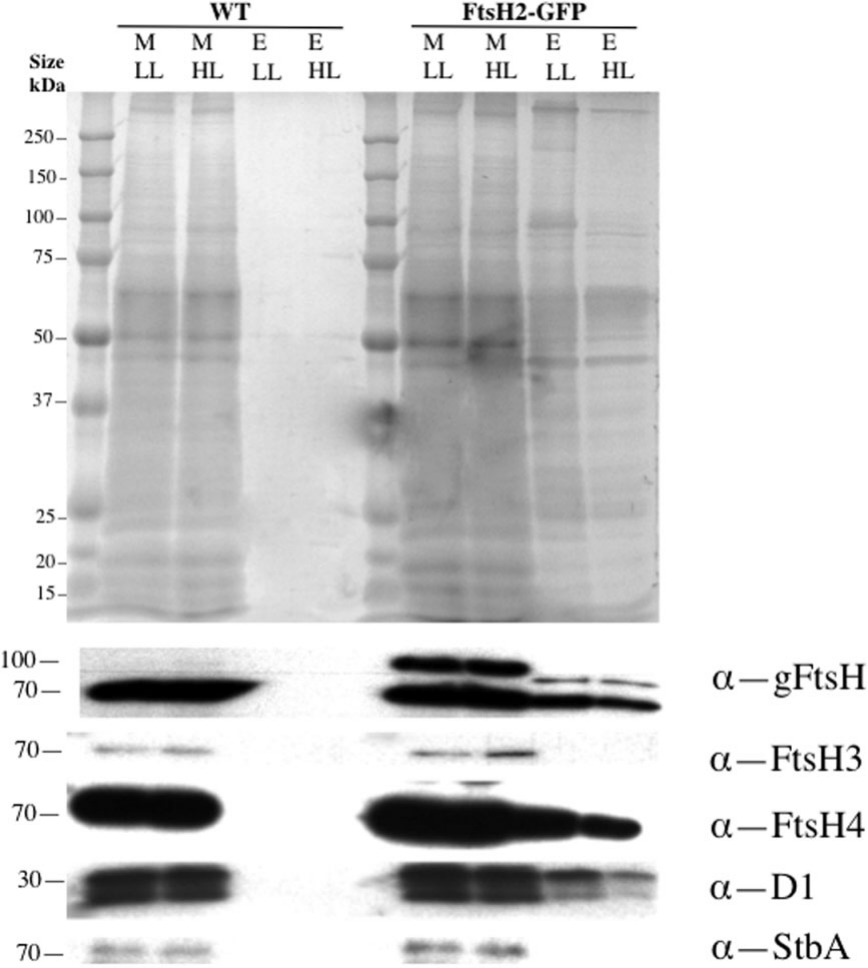
Effects of high‐light pre‐treatment on anti‐GFP pull‐downs from membranes of *S* *ynechocystis* ftsH2–gfp cells (wild‐type cells used as control). M: crude membrane fraction (sample containing 0.1 nmol chlorophyll loaded per lane); E: final elute of bound material, each lane containing the bound fraction from material containing 0.2 nmol chlorophyll loaded onto the column. Silver‐stained gel and immunoblots with antibodies to global FtsH, FtsH3, FtsH4, D1 and the plasma membrane marker StbA.

Our results from GFP tagging of FtsH2 indicate lateral heterogeneity of *Synechocystis* thylakoid membranes, with FtsH2 concentrated in distinct membrane zones and showing a very different distribution from that of chlorophyll (Fig. 3A, Fig. S6A). As an initial approach to investigating the composition of these FtsH2‐enriched thylakoid membrane zones, we used affinity pull‐downs based on magnetic beads functionalised with anti‐GFP antibody, as previously employed to examine the interactions of the Vipp1 protein (Bryan *et al*., 2014). Cells were disrupted by vortexing with glass beads, and a crude membrane preparation isolated by centrifugation. Membrane concentrations were standardised according to chlorophyll content, and the membrane suspensions were mixed with the functionalised magnetic beads and then loaded onto a minicolumn in a magnetic field, resulting in retention of the magnetic beads and their bound cargo. The bound fraction was washed and then eluted with detergent for protein analysis. The membranes were not detergent treated prior to loading onto the columns, but were washed with a buffer containing a low concentration of detergent (details in Experimental procedures section). As the GFP tags are attached to membrane‐integral proteins, the bound cargo most probably consists of membrane fragments. Work on *Gloeobacter violaceus* gives a precedent for the fractionation of a cyanobacterial membrane into functionally distinct zones following simple mechanical fragmentation (Rexroth *et al*., 2011). As a control for non‐specific retention on the columns, we loaded membranes from wild‐type cells lacking the GFP tag. All samples were standardised on the basis of the chlorophyll content of the material initially loaded onto the column. Sodium dodecyl sulfate–polyacrylamide gel electrophoresis (SDS–PAGE) gels (Fig. 6A) show that very little material from the wild‐type cells was retained on the column, whereas with *ftsH2–gfp* cells, a significant fraction of the loaded material was retained on the column until the final denaturing elution. The polypeptide profile of the bound fraction is significantly different from that of the crude membrane preparation (Fig. 6). Western blots show that the bound material includes FtsH2–GFP as expected (Fig. 6B and C), plus at least one other untagged FtsH protein recognised by a global anti‐FtsH antibody (Fig. 6D). A high proportion of the D1 protein is also retained on the column (Fig. 6E). In all these cases, retention of the protein occurs specifically in the *ftsH2–gfp* cells (Fig. 6B–E), indicating that it occurs through a direct or indirect association with FtsH2–GFP. The retained polypeptide profile changes strikingly if cells are exposed to HL prior to disruption, with stronger representation of a subset of membrane proteins (Fig. 7). Western blots show that somewhat less D1 protein and FtsH2–GFP were retained under these conditions (Fig. 7).

To further characterise the bound fraction, we identified polypeptides by mass spectrometry (summarised in Table 1; further details in Table S1). In the bound membrane fraction from LL *ftsH2–gfp* cells, we could identify a total of 23 proteins above threshold (excluding a small number of proteins also detected in the wild‐type sample and therefore likely to be non‐specific contamination) (Table 1). The retained proteins include components of both photosystems, and the proton‐translocating ATP'ase. Apart from FtsH2 itself, FtsH4 was the only other FtsH protein detected above threshold (Table 1). We cannot exclude the presence of FtsH3, which has previously been shown to form a hetero‐oligomer with FtsH2 (Boehm *et al*., 2012), but any FtsH3 present must be below the detection threshold. The *slr1128* gene product was detected by mass spectrometry: this Band 7 stomatin‐like protein was reported to be important for survival of *Synechocystis* under HL and is a potential interaction partner for the HLIP proteins that stabilise PSI trimers (Wang *et al*., 2008), although there are conflicting results (Boehm *et al*., 2009).

**Table 1:** Protein identification from mass spectrometry

ORF	Protein	Protein role	*ftsH2–gfp* LL	*ftsH2–gfp* HL	WT
*slr0335*	ApcE	Phycobiliprotein ApcE	✓	–	✓
*slr1311*	CcmM	Carbon dioxide concentrating mechanism protein	✓	–	✓
*slr0012*	RbcL	Ribulose bisphosphate carboxylase large chain	–	–	✓
*slr1834*	PsaA	Photosystem I P700 chlorophyll a apoprotein A1	✓	✓	–
*slr1835*	PsaB	Photosystem I P700 chlorophyll a apoprotein A2	✓	✓	✓
*ssl0563*	PsaC	Photosystem I iron–sulfur center OS	✓	–	–
*slr0737*	PsaD	Photosystem I reaction center subunit II	✓	✓	–
*ssr2831*	PsaE	Photosystem I reaction center subunit IV	✓	–	–
*smr0004*	PsaI	Photosystem I reaction center subunit VIII	✓	–	–
*slr1655*	PsaL	Photosystem I reaction center subunit XI	✓	–	–
*sll0851*	CP43	Photosystem II 44 kDa reaction center protein	✓	✓	–
*slr0906*	CP47	Photosystem II CP47 chlorophyll apoprotein	✓	✓	–
*ssl2598*	PsbH	Photosystem II reaction center protein H	✓	–	–
*ssr3451*	PsbE	Cytochrome b559 subunit alpha	✓	–	–
*sll0849*	PsbD	Photosystem II D2 protein	–	✓	–
*slr1311*	PsbA2	Photosystem Q(B) protein (D1)	✓	✓	–
*slr1326*	AtpA	ATP synthase subunit alpha	✓	–	–
*slr1329*	AtpB	ATP synthase subunit beta	✓	–	–
*slr1327*	AtpC	ATP synthase gamma chain	✓	–	–
*slr0228*	FtsH2	ATP‐dependent zinc metalloprotease FtsH2	✓	✓	–
*sll1463*	FtsH4	ATP‐dependent zinc metalloprotease FtsH4	✓	–	–
*sll0247*	IsiA	Iron stress‐induced chlorophyll‐binding protein	✓	–	–
*sll1578*	CpcA	C‐phycocyanin alpha chain	✓	–	–
*slr1128*	Hypothetical	Band 7 protein, stomatin homologue	✓	–	–

Comparison of proteins detected above threshold in anti‐GFP affinity pull‐downs of *Synechocystis ftsH2–gfp* cells in LL and following HL exposure, with wild‐type (low light) control. Samples were isolated from membranes containing equal chlorophyll. Two biological replicates were tested for each strain and condition.

The mass spectrometric results show that HL treatment induces quantitative changes in the bound polypeptide profile, as also indicated by the SDS–PAGE gels (Fig. 7). Following HL exposure, a smaller set of polypeptides is detected above threshold, including some core PSII and PSI proteins but lacking the proton‐translocating ATP'ase and several minor photosystem subunits (Table 1).

## Discussion

Here we have constructed and visualised strains of *Synechocystis* with C‐terminal GFP tags on the 4 FtsH proteins. To avoid over‐expression artefacts, the tagged FtsH proteins were expressed from their native chromosomal loci, under the control of their native promoters. FtsH1–3 appeared fully functional; with FtsH4, we cannot be sure if function is retained as the null mutant has a similar phenotype to the wild type (Mann *et al*., 2000). It can never be absolutely excluded that GFP tagging perturbs the localisation of the protein, but our results show that PSII repair is not measurably affected by tagging of FtsH2 with GFP. Therefore, it is very unlikely that FtsH2 localisation is perturbed. Similarly, GFP tagging of respiratory electron transport complexes in the cyanobacterium *Synechococcus* sp. PCC7942 had no measurable effect on electron transport function, although these complexes are highly localised under some conditions (Liu *et al*., 2012). The formation of inclusion bodies is unlikely as the proteins are functional and not over‐expressed, and the fluorescence images (Figs 2, 3, 4, 5) suggest location in the thylakoid or cytoplasmic membranes rather than in inclusion bodies. In the case of FtsH2, the biochemical data from affinity pull‐downs (discussed below) strongly confirm location in the thylakoid membrane.

The sub‐cellular locations of the FtsH proteins are broadly consistent with previous biochemical studies. FtsH1 is observed mainly in the cytoplasmic membrane, consistent with Pisareva *et al*. (2007), but with a possible smaller sub‐population in the thylakoid membrane. FtsH2 appears entirely in the thylakoid membrane, as previously observed (Komenda *et al*., 2006). FtsH3 and FtsH4 both appear concentrated at the distal edge of the thylakoid membrane adjacent to the cytoplasmic membrane in LL, but their distributions shift inwards away from the cell periphery after HL exposure. Biochemical fractionation has previously indicated that FtsH3 is found in the cytoplasmic membrane (Pisareva *et al*., 2007). The radial distribution of FtsH3–GFP extends beyond the thylakoid membranes in LL (Fig. 1C), suggesting a sub‐population in the cytoplasmic membrane. This would be consistent with an FtsH1/FtsH3 hetero‐oligomer in the cytoplasmic membrane (Krynická *et al*., 2014). FtsH proteins can form stable hetero‐oligomers and affinity pull‐downs indicate hetero‐oligomers of both FtsH2/FtsH3 and FtsH1/FtsH3 (Boehm *et al*., 2012). Our results from fluorescence microscopy suggest that both hetero‐oligomers would have to be located in LL at the distal edge of the thylakoid membrane system or at an interface between the thylakoid and cytoplasmic membranes, where the distribution of FtsH3 overlaps with those of FtsH2 and FtsH1, although comparison between strains might not be straightforward if there are effects of GFP tagging on the assembly and accumulation of the various FtsH complexes. The inward shift in the radial distribution of FtsH3 after HL exposure (Fig. 4B) makes its distribution more similar to that of FtsH2 (Fig. 3B). This could allow increased formation of an FtsH2/FtsH3 hetero‐oligomer under these conditions; however, direct evidence for an increase in such a hetero‐oligomer under HL is lacking. The overall distributions of FtsH1–3 are distinct, especially under LL, suggesting that these proteins could be present as monomers or homo‐oligomers as well as hetero‐oligomers. Previous studies in plants and cyanobacteria do not rule out this possibility (Sakamoto *et al*., 2003; Boehm *et al*., 2012).

All of FtsH1–4 show distinctly patchy distributions in their respective membranes, indicating a tendency to segregate into localised membrane zones. The three thylakoid‐membrane FtsHs (FtsH2–4) show membrane sub‐localisation that is clearly distinct from that of chlorophyll, as judged from chlorophyll fluorescence images. This patchy distribution of membrane‐integral proteins is evidence for extensive lateral heterogeneity in cyanobacterial thylakoid membrane composition, as is also apparent from the patchy distributions of respiratory complexes in *Synechococcus* sp. PCC7942 (Liu *et al*., 2012). FtsH3 and FtsH4 are both concentrated in patches at the distal edge of the thylakoid membrane system, adjacent to the cytoplasmic membrane. This matches the presumed distribution of Photosystem II assembly centres, which are postulated to be located at the distal edge of the thylakoid membrane system and in contact with the cytoplasmic membrane and periplasm (Schottkowski *et al*., 2009; Stengel *et al*., 2012). It will be interesting to test the possibility that FtsH3 and/or FtsH4 co‐localise with other components of these assembly centres such as PratA (Schottkowski *et al*., 2009; Stengel *et al*., 2012).

We focused on the location and interactions of FtsH2 because of the strong evidence for its direct involvement in the Photosystem II repair cycle (Silva *et al*., 2003; Komenda *et al*., 2006). As previously reported, FtsH2 is found exclusively in the thylakoid membranes where it has a distribution distinct from that of chlorophyll (Komenda *et al*., 2006; Krynická *et al*., 2014). In our experiments, FtsH2 was localised in distinct patches with a mean diameter of about 370 nm, and around three to eight patches per cell, but it is possible that the details of FtsH2 distribution depend on the exact strain and growth conditions. We found that FtsH2 retains its patchy distribution after HL exposure. HL incubation sufficient to trigger the PSII repair cycle had no quantifiable effect on the sub‐cellular distribution of FtsH2. This implies that the localised FtsH2‐enriched thylakoid membrane zones are the location of Photosystem II repair activity, or at least of the crucial early step in which damaged D1 subunits are removed and degraded by FtsH2 (Silva *et al*., 2003). As a first approach to identifying other constituents of the FtsH2‐defined membrane zones, we used anti‐GFP affinity pull‐downs on *ftsH2–gfp* cells that had been mechanically broken but not detergent treated prior to loading. Subsequent washes used low concentrations of detergent (see details in Experimental procedures section). The aim was to pull down membrane fragments corresponding to FtsH2‐enriched thylakoid membrane zones observed *in vivo*. We found that a distinctive membrane fraction was pulled down only from *ftsH2–gfp* cells and not from the wild‐type control, indicating that it was pulled down as a consequence of direct or indirect association with FtsH2–GFP. The membrane fraction included numerous thylakoid membrane components, but did not contain the cytoplasmic membrane marker SbtA (Norling *et al*., 1998) at detectable levels. Although the pull‐downs clearly contain thylakoid membrane components, their composition is quantitatively different from that of the crude membrane preparation. The protein profile is also significantly altered by HL exposure that activates the PSII repair cycle, with a smaller set of components detected above threshold by mass spectrometry. Thus, it appears that the population of photosynthetic membrane proteins in proximity to FtsH2 changes according to light intensity, with a higher proportion of the core components of the photosynthetic reaction centres present when the repair cycle is active. Surprisingly, and in contrast to other reaction centre core components, the proportion of the D1 protein retained on the column is greater in LL, when it can account for a considerable fraction of the D1 in the cell (Figs 6 and 7). In addition to photosynthetic proteins and FtsH2 itself, we could also detect FtsH4 and Slr1128. Slr1128 is a Band 7 stomatin‐like protein reported to be important for survival of *Synechocystis* under HL and to be a potential interaction partner for the HLIP proteins that stabilise PSI trimers and protect cells under HL (Wang *et al*., 2008), although there are conflicting results concerning its membrane localisation and role (Boehm *et al*., 2009). Our failure to detect FtsH3 above threshold in the pull‐downs is surprising in view of convincing evidence for an FtsH2/FtsH3 hetero‐oligomer involved in PSII repair (Boehm *et al*., 2012). However, not all FtsH2–GFP is retained on the column (Fig. 7), and it is possible that FtsH2/FtsH3 complexes might not have bound as effectively as FtsH2 homo‐complexes. There is also a strain difference between the two studies, since Boehm *et al*. (2012) used the glucose tolerant strain of *Synechocystis*, which has some significantly different properties to the PCC6803 strain used here (Kanesaki *et al*., 2012). In addition, more detailed biochemical characterisation of the FtsH2–GFP subunit isolated in the pull‐downs is required to confirm that it is a component of a hexameric FtsH complex. Nevertheless, our results do raise the possibility that the composition of FtsH oligomers might show more plasticity than previously thought.

Our FtsH2–GFP pull‐downs provide a starting point for further investigation of the composition of the putative FtsH2‐defined repair zones in the thylakoid membrane. Our results suggest that these membrane zones have a complex and dynamically variable composition, and a full understanding of their role will require investigation of the localisation and interactions of other components in addition to FtsH2. However, the methodology used here provides a first step towards identifying other proteins that may be active in these areas of the membrane, and shows promise as a general route for the further investigation of membrane lateral heterogeneity initially identified by fluorescent protein tagging and microscopy.

If FtsH2 remains in its localised thylakoid membrane domains when the PSII repair cycle is active, this implies that photodamaged PSII centres must migrate to FtsH2 to be repaired, rather than FtsH2 circulating through the bulk thylakoid membrane to repair PSII *in situ*. In green plant chloroplasts, photodamaged PSII reaction centres must migrate from the grana to the stroma lamellae or the grana margins to be repaired (Aro *et al*., 1993; Yoshioka *et al*., 2010). It is interesting that cyanobacteria also appear to maintain spatial separation of PSII repair activity from the bulk of active PSII centres, even though they lack membrane appression and grana stacking. In green plants, the protein kinases STN7 and STN8 are important for mobilisation of Photosystem II, allowing diffusion of PSII centres out of the grana for repair in the stroma lamellae (Bonardi *et al*., 2005; Goral *et al*., 2010). There are no obvious homologues of these kinases in cyanobacteria (Allahverdiyeva *et al*., 2013). However, it appears that similar mobilisation of Photosystem II must be necessary in cyanobacteria, since fluorescence recovery after photobleaching measurements show that PSII centres are almost completely immobile in the membrane under normal conditions (Mullineaux *et al*., 1997; Sarcina and Mullineaux, 2004). PSII diffusion is difficult to quantify in *Synechocystis* because of the irregular shape of the thylakoid membranes (Mullineaux and Sarcina, 2002). However, a study in the cyanobacterium *Synechococcus* sp. PCC7942 showed that a proportion of chlorophyll fluorescence (likely from PSII reaction centres) does indeed become mobile following exposure to intense red light (Sarcina *et al*., 2006). Furthermore, these conditions induce partial redistribution of chlorophyll fluorescence into distinct regions (Sarcina *et al*., 2006), which could be the equivalents in *Synechococcus* of the FtsH2‐defined membrane zones that we observed in *Synechocystis*. Therefore, it is likely that cyanobacteria possess mechanisms to facilitate the diffusion of PSII under photo‐damaging conditions, allowing damaged PSII centres to visit specialised repair zones in the membrane. The biochemical mechanism of PSII mobilisation in cyanobacteria remains to be established.

Given the problems caused by restricted mobility of PSII reaction centres in the membrane, what advantage could be served by segregating repair activity into distinct membrane zones? The bulk thylakoid membrane is densely packed with photosynthetic reaction centres and its cytoplasmic surface is largely covered with phycobilisomes (Grossman *et al*., 1993; Mullineaux, 1999). It may therefore be impossible to establish a sufficient concentration of repair factors in the bulk membrane to allow the repair process to proceed efficiently, necessitating the segregation of the repair cycle into distinct membrane zones. A second, and not mutually exclusive, possibility relates to the damaging effects of reactive oxygen species (ROS) on PSII repair, and specifically on translation of new protein subunits (Murata *et al*., 2012). Active PSII is a potent source of ROS (Krieger‐Liszkay, 2005). ROS species diffuse rapidly but are also rapidly quenched in the cytoplasm, giving them limited diffusion range (Skovsen *et al*., 2005; Inoue *et al*., 2011). Singlet oxygen has a range in the order of 200–300 nm when diffusing in the cytoplasm (Skovsen *et al*., 2005). In a cell as small as *Synechocystis*, it will clearly be impossible to completely avoid ROS generated by active PSII, but nevertheless there must be significant variation in ROS concentration within the cell, on 100 nm scales. Segregating repair zones away from active PSII could serve to reduce the damaging effects of ROS on PSII repair.

## Experimental procedures

### Strains and growth

*Synechocystis* sp. PCC 6803 (not the glucose tolerant strain) was grown in BG11 medium (Castenholz, 1988), at 30°C and low white light at 7 μE m^−2^ s^−1^ in a shaking incubator. Transformants were grown in the presence of 50 μg ml^−1^ apramycin. Cells for all experiments were taken at early stationary phase.

### High‐light treatment

*Synechocystis* sp. PCC 6803 strains previously grown in LL were exposed for 1 h to white light at 600 μE m^−2^ s^−1^ in a custom‐built light box with water cooling to maintain the cells at room temperature. Where specified, lincomycin was added to 400 μg ml^−1^ prior to HL exposure to inhibit protein synthesis.

### Construction of transformants expressing GFP fusions

Transformants expressing C‐terminal eGFP fusions to FtsH1 (slr1390), FtsH2 (slr0228), FtsH3 (slr1604) and FtsH4 (sll1463) (all from the native chromosomal loci) were generated using a modified REDIRECT method (Gust *et al*., 2003).

Each target gene with its 1 kb flanking regions was amplified from the wild‐type *Synechocystis* sp. PCC 6803 genome by polymerase chain reaction (PCR) with the following forward (F) and reverse (R) primers: *slr1390*F: 5′‐TTCCGCGAAGCTCTAGTCAC‐3′, *slr1390*R: 5′‐CGATGTCGTTGCGGACATGG‐3′, *slr0228*F: 5′ACCATTGTGGCTCCGCCATC‐3′, *slr0228*R: 5′‐TCGGTTGCGTGCGCTTACTG‐3′, *slr1604*F: 5′‐CATCGCGGTCAATTCCAAGT‐3′, *slr1604*R: 5′‐CCATTACCGTGGCGGAATTA‐3′, (*sll1463*F: 5′‐TTCACCACCGACTCCTATGT‐3′, *sll1463*R: 5′‐CATCGGAGTTGGAGCCAGAA‐3′. These 3–4 kb PCR products were cloned into pGEM T‐easy vector (Promega) and the plasmids were used to transform *E. coli* strain BW25113.

Two long PCR primers were used to amplify the *gfp*‐apramycin^r^ cassette flanked by flipase recognition target sites from the pIJ786 plasmid (strain and plasmid provided by PBL Biomedical Laboratories). Each long primer has at the 5′ end 39nt matching *Synechocystis* sequence either side of but not including the stop codon and a 3′ sequence (19 nt or 20 nt) matching the right or left end of the cassette. The product was introduced by electroporation into *E. coli* BW25113, where homologous recombination results in incorporation of *gfp* and the 20 nt linker region at the 3′ end of each *ftsH* gene, with the apramycin resistance marker downstream (Supporting Fig. S1A). Transformants were selected by resistance to both ampicillin and apramycin. Plasmids were screened by PCR and then sequenced.

### Transformation of *S* *ynechocystis* sp. PCC 6803

*Synechocystis* sp. PCC 6803 cells were transformed with plasmid DNA according to Chauvat *et al*. (1989), spread onto BG‐11 agar plates and incubated in white light at 50 μE m^−2^ s^−1^ at 30°C until confluent green growth was observed. Apramycin 50 μg ml^−1^ was then added to 50 μg ml^−1^ and the plates were incubated as before. Surviving single colonies were then transferred to liquid BG‐11 media with 50 μg ml^−1^ apramycin. Cultures were grown and transferred to fresh antibiotic containing media three to five times. Genomic DNA from the transformants was extracted (ZR Fungal/Bacterial DNA MiniPrep, Zymo Research) and correct integration of the construct and complete segregation were confirmed by PCR (Supporting Fig. S1).

### Oxygen evolution measurements

Oxygen evolution was measured at 30°C in a Clarke‐type oxygen electrode (OxyLab2, Hansatech, King's Lynn, UK) at saturating light intensity (white light at 1000 μE m^−2^ s^−1^). Cell suspensions were adjusted to a chlorophyll *a* concentration of 10 μM (chlorophyll measured according to Komenda and Barber, 1995). Measurements were performed in the presence of an artificial PSII electron acceptor [1 mM 2,6‐dichloro‐*p*‐benzoquinone kept oxidised with 3 mM potassium ferricyanide] to ensure that oxygen evolution is not limited by downstream electron transport. Under these conditions, oxygen evolution is a direct indicator of the level of functional PSII (Graan and Ort, 1986).

### SDS–PAGE and immunoblotting

Membranes were isolated from *Synechocystis* sp. PCC 6803 strains by mechanical cell breakage and differential centrifugation. For each strain, a 15 ml liquid culture was grown under LL or HL treated. Culture densities were normalised according to its OD_730_ and the cells were harvested, re‐suspended and washed twice in ACA buffer [750 mM ε‐amino caproic acid, 50 mM BisTris/HCl pH = 7.0, 0.5 mM ethylenediamenetetraacetic acid (EDTA)]. The final volume of cell suspension was 500 μl to which 200 μl of glass beads (212–300 μm in diameter, Sigma‐Aldrich, UK) were added. Cells were broken with a vortexer at 4°C using a 1 min on/1 min off cycle repeated three to five times. Crude membranes were prepared by differential centrifugation (Komenda *et al*., 2002) and resuspended in 50 μl KPN buffer (40 mM K‐phosphate, pH = 8.0, 100 mM NaCl) supplemented with Protease Inhibitor Cocktail (Roche). Chlorophyll *a* content was determined by measuring its absorption at 666 and 750 nm in methanol (dilution 1:200) (Komenda and Barber, 1995). Suspensions of crude membranes were either used for anti‐GFP tagged affinity pull‐downs or for SDS–PAGE and immunoblotting. Prior to SDS–PAGE, samples were incubated for 30 min in Laemmli 2 × SDS sample buffer containing 5% β‐mercaptoethanol. Gels were electro‐blotted onto nitrocellulose membrane using the iBlot system (Invitrogen, UK) according to the manufacturer's instructions. Immunoblotting analysis was performed using specific primary antibodies as listed: α‐GFP antibody supplied by Gentaur Molecular Products, Belgium; α‐gFtsH: polyclonal antibody raised against residues 297–312 of *E. coli* FtsH (Tomoyasu *et al*., 1993), which potentially cross‐reacts with all *Synechocystis* sp. PCC 6803 FtsH homologues, kindly provided by Teru Ogura (University of Kumamoto, Japan); α‐FtsH1: polyclonal antibody raised against residues 578–592 of *Synechocystis* FtsH1, α‐FtsH2 against residues 98–115 of *Synechocystis* FtsH2, α‐FtsH3 against residues 59–75 of *Synechocystis* FtsH3, all at 1 in 1000 dilution, and αFtsH4 against residues 556–574 of *Synechocystis* FtsH4, at 1 in 10,000 dilution; α‐D1: a rabbit polyclonal antiserum (#304‐F) raised against residues 325–353 of precursor D1 from pea (*Pisum sativum*; Nixon *et al*., 1990) at a dilution of 1 in 5000; α‐SbtA: rabbit polyclonal antiserum raised against residues 184–203 of *Synechocystis* SbtA, dilution 1:200,000 (kindly provided by T. Ogawa). Secondary anti‐rabbit/anti‐mouse antibodies were horseradish peroxidase‐conjugated (GE Healthcare). Signals were visualised using a chemiluminescent kit (GE Healthcare).

### Anti‐GFP affinity pull‐downs

One hundred microlitres of prepared crude *Synechocystis* membranes (chlorophyll *a* at 10 μM) was incubated with 50 μl of α‐GFP MicroBeads rotating for 1 h at 4°C. Minicolumns (μMACS Epitope Tag Protein Isolation Kit) were placed into the magnetic field stand, washed with supplied lysis buffer, following the wash with Buffer B. One hundred fifty microlitres of membrane mixture was loaded onto the column and the flow‐through fraction collected. The column was washed six times with 200 μl Wash 1 Buffer (150 mM NaCl, 1% Igepal CA‐630, 0.5% sodium deoxycholate, 0.1% SDS, 50 mM Tris–HCl pH 8.0), twice with 100 μl of Wash 2 Buffer (20 mM Tris–HCl pH 7.5) and finally the bound fraction was eluted with elution buffer (50 mM Tris–HCl pH 6.8, 50 mM dithiothreitol, 1% SDS, 1 mM EDTA, 0.005% bromophenol blue, 10% glycerol) at 95°C. All fractions were collected and concentrated using centrifugal filter concentrators (Vivaspin, cut‐off 10 kDa) to final volumes of 50 μl. Samples were analysed by SDS–PAGE and mass spectrometry, as will be described later.

### MALDI TOF/TOF MS

Samples in elution buffer containing 0.005% bromophenol blue were run a short distance not sufficient for protein separation (1–1.5 cm) into 10% Mini Protein precast gels. Immediately following electrophoresis, protein bands were excised from the gel with a clean sharp razor. The resulting blue‐tinted gel slices had dimensions of 1.5 mm × 5 mm. Samples were obtained from two biological replicates for each strain and condition.

In preparation for mass spectrometry, samples were digested with trypsin (E.C.3.4.21.4, Promega) overnight. Tryptic peptides were separated on an offline Ultimate 3000 nanoLC system using a PepMap 100 75 mm × 15 cm fused silica C18 analytical column (Dionex), coupled to a Probot for fraction collection and matrix addition with α‐cyano‐4‐hydroxycinnamic acid as the matrix. A gradient of 2–60% ACN (acetonitrile) in 0.1% trifluoroacetic acid was delivered over 60 min at a flow rate of 0.300 nl min^−1^. Matrix‐assisted laser desorption and ionisation time of flight (MALDI TOF)/TOF mass spectrometry (MS) was performed using a AB Sciex 4800 mass spectrometer (Foster City, CA) in the positive reflectron mode with delayed extraction. MS precursor acquisition was followed by interpretation and data‐dependent MS/MS acquisition with the CID (collision‐induced dissociation) on. Data interpretation was configured to select a maximum of 10 precursor ions per fraction with a minimum signal‐to‐noise ratio of 50. The data were processed using GPS Explorer (Applied Biosystems, CA) against Swiss‐Prot and Cyanobase databases. Search parameters were enzyme = trypsin: fixed modifications = carboxymethyl (C); variable modifications = oxidation (M); mass tolerance ± 100 ppm: fragment mass tolerance = 0.3 Da: maximum missed cleavages = 1; mass values = monoisotopic.

### Confocal microscopy

Drops of cell culture were adsorbed onto the surface of BG11 agar plates to immobilise the cells, and blocks of agar with the cells on the surface were mounted under a glass cover slip in a custom‐built sample holder. Fluorescence micrographs were recorded with a Leica TCS‐SP5 laser‐scanning confocal microscope with a 63× oil immersion objective (numerical aperture, 1.4). Excitation was at 488 nm from an argon laser. The confocal pinhole was set to give resolution in the *z‐*direction of ∼ 1 μm. GFP and chlorophyll fluorescence emission were collected at 502–512 nm and 670–720 nm respectively (wavelength ranges selected by monochromators). All images were recorded at 12‐bit resolution (512 × 512 pixels), with laser scanning at 400 Hz and 2× line averaging. GFP fluorescence was distinguished from background autofluorescence by recording images before and after a laser exposure that selectively bleaches GFP fluorescence (Komenda *et al*., 2006; Liu *et al*., 2012). After recording a pre‐bleach image, the frame was scanned 10× with laser power increased by a factor of 2.4 to bleach GFP fluorescence. A post‐bleach image was then recorded. Clean GFP images were obtained by subtracting the post‐bleach image from the pre‐bleach image.

### Data analysis

Images were analysed using ImageJ software with MBF (McMac Biophotonics Facility) and JACoP plugins. In Fig. 1C and D, radial fluorescence profiles were determined for each cell by using the chlorophyll fluorescence to define the cell geometry, i.e. radial coordinates were used to allow cell fluorescence to be averaged over rotation angle in each cell. Images were segregated into cells using chlorophyll fluorescence to demarcate approximate cell boundaries. Averaging over cells was performed by rescaling to a standard 1/2 maximum radius, filtering out cells that were not sufficiently a circular (i.e. undergoing cell division). A bleached image was used to calibrate the auto fluorescence, i.e. levels of GFP protein were defined as relative to the bleached image. In Figs 2, 3, 4, 5, a circular region of interest was defined manually for each cell, and a radial distribution was obtained by summing the fluorescence values at each given distance from the centre. Distributions were then standardised to the cell radius. This analysis used a radial profile plot written by Paul Baggethun for ImageJ.

## Supporting information

Supporting Information
