# Supplementary material for: Sub‐cellular location of FtsH proteases in the cyanobacterium S ynechocystis sp. PCC 6803 suggests localised PSII repair zones in the thylakoid membranes
Source: Mol Microbiol. 2015 Feb 11;96(3):448–62. doi: 10.1111/mmi.12940 (PMC4949578; doi:10.1111/mmi.12940)
Supplement: Supplementary file 1 — Supporting Information [file MMI-96-448-s001.pdf]

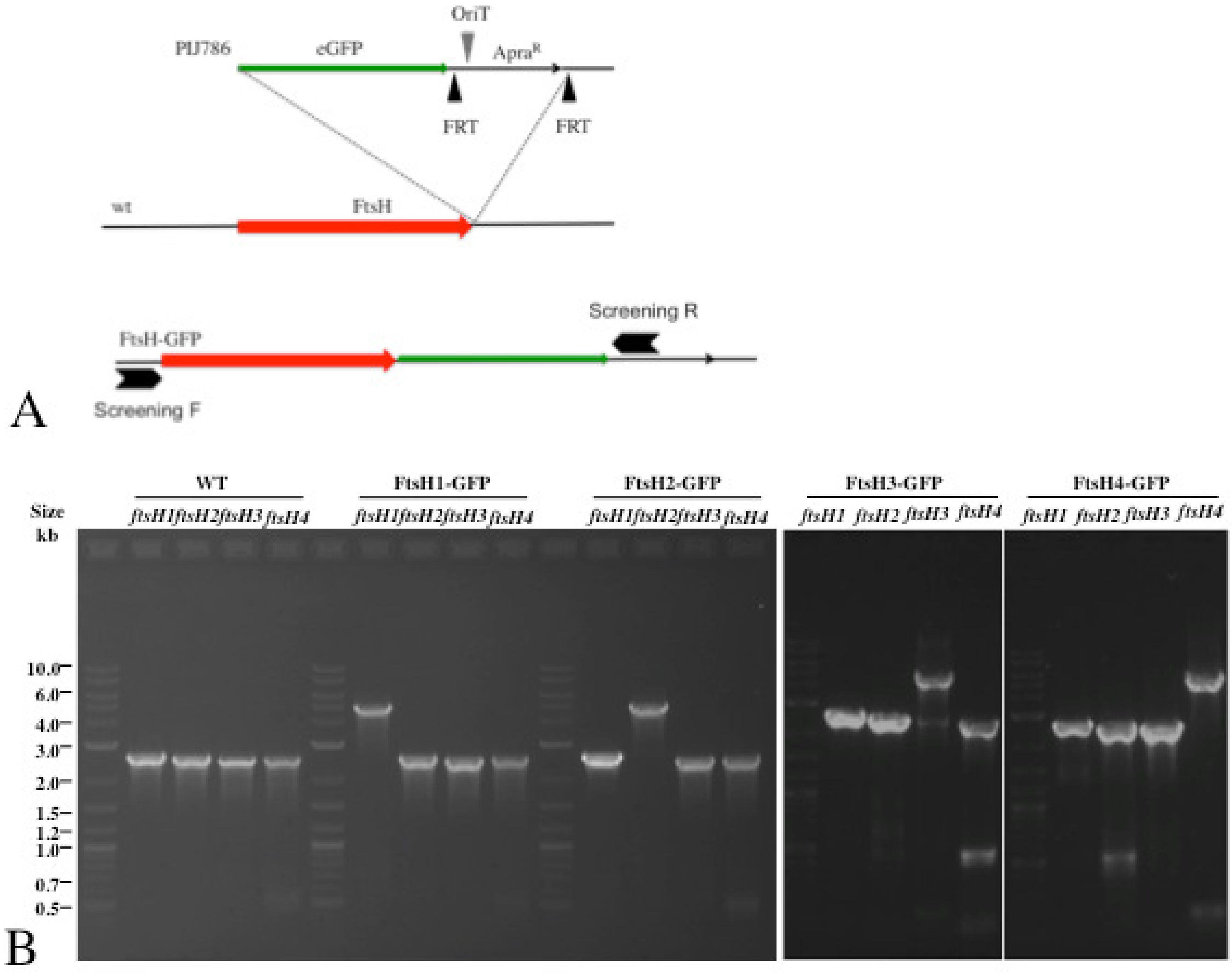

Supporting Figure 1 (A) Construction of genes encoding GFP fusions to the C-terminal end of each FtsH protease by amplification of wild-type gene and *gfp-Apramycin*^{R} cassette from plasmid PIJ786. (B) segregation test and screening for *gfp* fusion with PCR. Primers chosen to amplify the entire *ftsH* ORF and 650bp downstream sequence. Expected sizes of PCR products ~2.5kb for wild-type and ~4.5kb for *ftsH+gfp+Apra*^{R}.

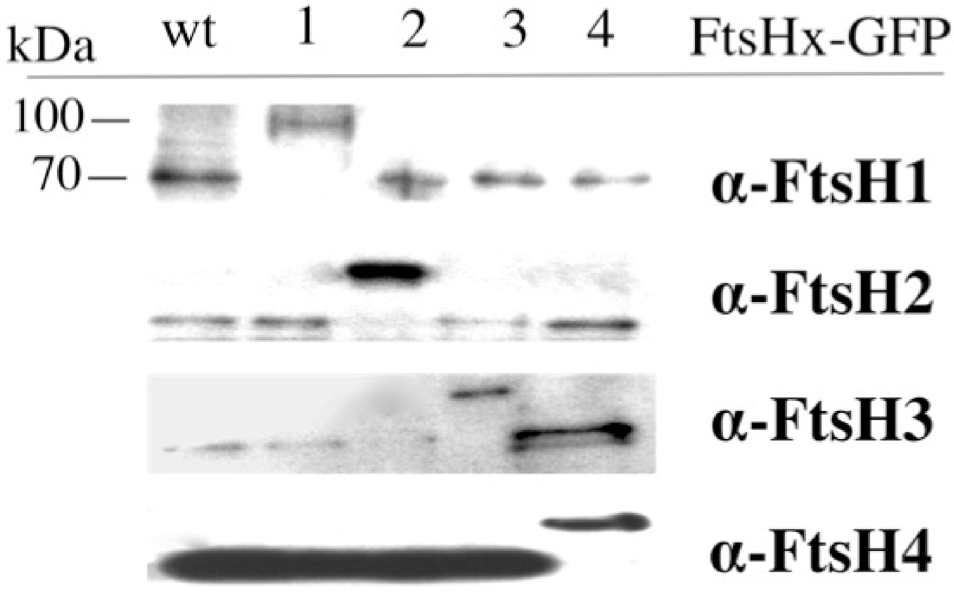

Supporting Figure 2 Immunoblots with crude membrane fractions isolated from wild-type and each of the GFP-tagged strains (1 = *ftsH1-gfp*; 2 = *ftsH2-gfp*; 3 = *ftsH3-gfp*; 4 = *ftsH4-gfp*). Immunoblots performed with anti-FtsH antibodies specific for each FtsH protein. GFP-tagged FtsH proteins are ~100 kDa; native FtsH proteins ~70 kDa. Lanes loaded according to chlorophyll concentration (0.1 nmol chlorophyll per lane).

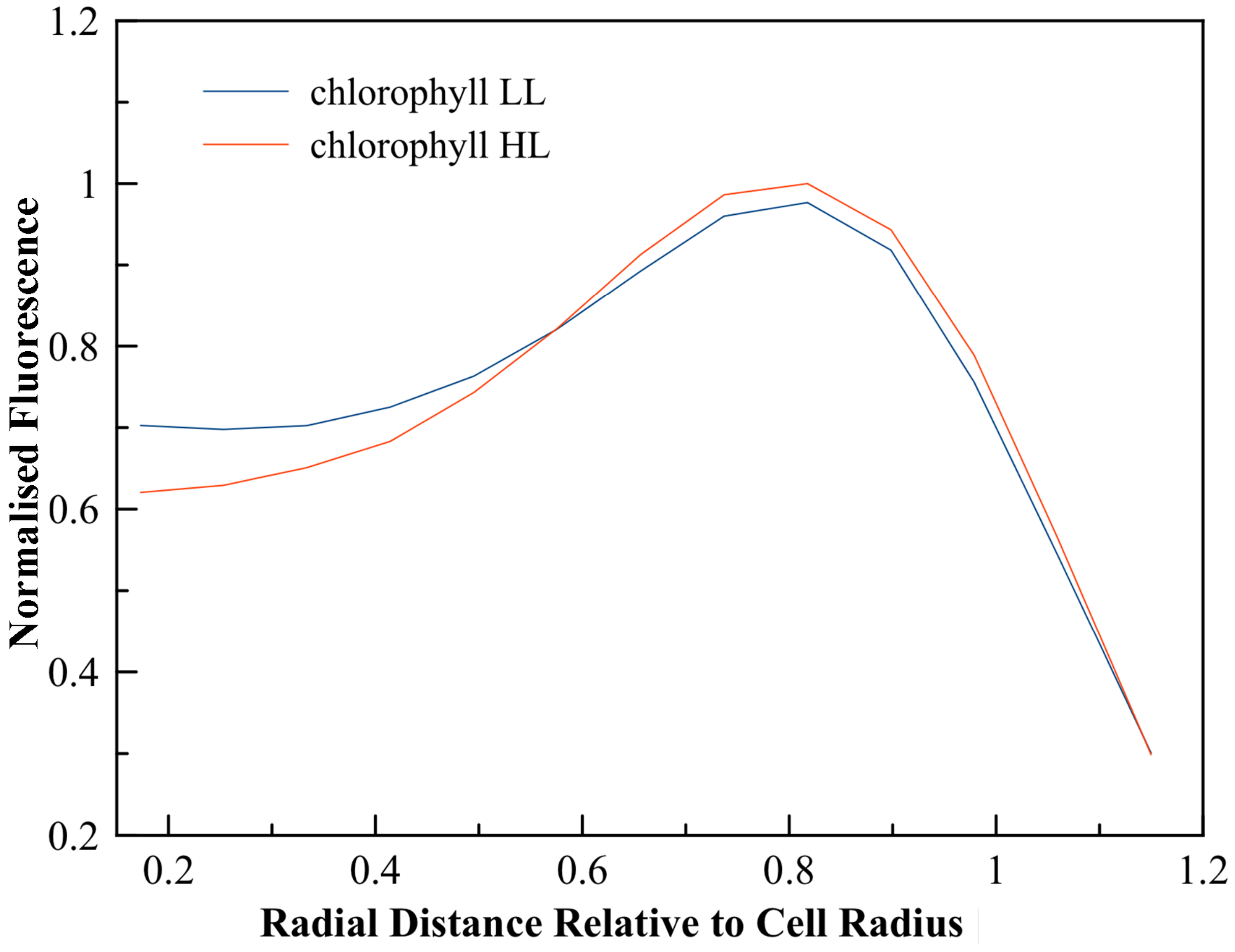

Supporting Figure 3 High light exposure has little effect on the radial distribution of chlorophyll fluorescence in *Synechocystis* cells. Wild-type cells were grown in low light (LL) or exposed to high light (HL) at 600 μE m^{-2} s^{-1} for 45-60 min. Averaged fluorescence radial distributions (n = 30, for 3 biological replicates), standardised to cell radius.

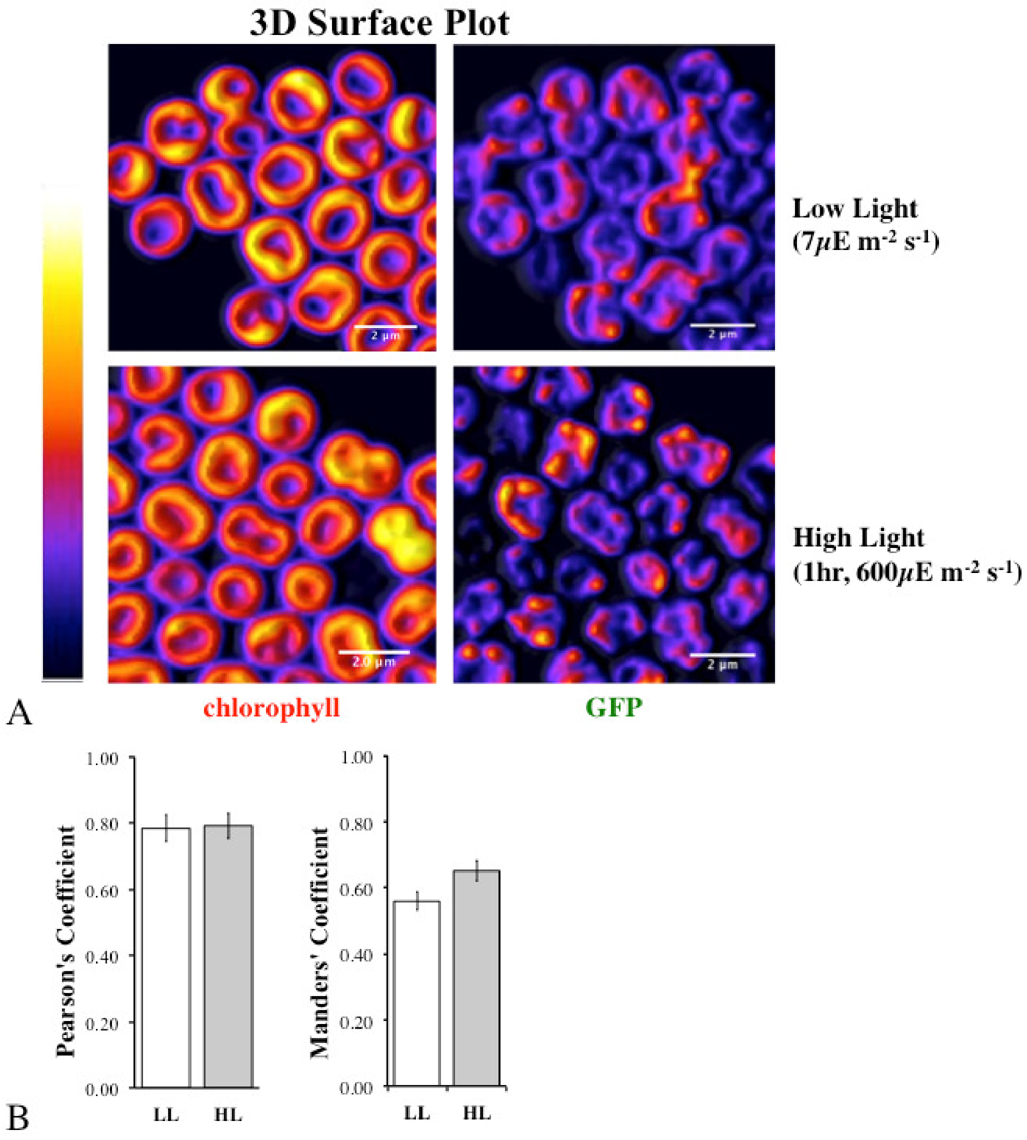

Supporting Figure 4 Effect of high-light exposure on the relative distributions of chlorophyll and FtsH2-GFP in *Synechocystis* cells (A) False-colour 3D surface plots (with linear colour scale) of GFP and chlorophyll fluorescence in low-light and high light-exposed *Synechocystis* cells (B) Co-localisation of GFP and chlorophyll fluorescence assessed by Pearson's and Manders' coefficients. There are no significant differences between HL and LL (n = 30, p = 0.30 for Pearson's coefficient and 0.26 for Manders' coefficient, error bars show standard deviation, tested and averaged for 3 biological replicates).

Supporting Table 1. Mass spectrometric analysis of proteins from anti-GFP pull-downs from *Synechocystis ftsh2-gfp* cells, showing recognised peptide sequences and ion scores, based on the calculated probability, P, Ion score cut off = 1. Sequences and ion scores are from low-light samples, except for proteins detected exclusively after high-light treatment (see Table 1). Data from 2 biological replicates for each condition.

ORF	Protein ID	Sequence	Ion score
slr0335	ApcE	FVELGQVSAIR	31
		AYSQSISYLESQVR	36
		RFPTLPAANFPNTER	6
		EIQQYNQILASQGLK	31
		EYSDAFGEDTVPYER	31
slr1311	CcmM	Carbon dioxide concentrating mechanism protein	✓
slr0012	RBL_SYN3	FLFVQEAIEK	51
		WSPELAAACELWK	17
slr1834	psaA	WGKPGHFDR	56
		LVPDKGQLGFR	41

		**DYDPAKNVNNLLDR**	**53**
slr1835	**psaB**	**FSQDLAQDPTTR**	**28**
		**DFGYSFPCDGPGR**	**27**
		**VYLGAETTR**	**28**
ssl0563	**psaC**	**CETACPTDFLSIR**	**72**
		**IYDTCIGCTQCVR**	**78**
		**ACPLDVLEMVPWDGCK**	**16**
slr0737	**psaD**	**IGQNPEPVTIK**	**51**
		**KEQCLALGTQLR**	**26**
		**VYPSGEVQYLHPADGVF PEKVNEGR**	**64**
smr0004	**psaI**	**Photosystem I reaction center subunit II**	
ssr2831	**psaE**	**SGILYPVIVR**	**50**
slr1655	**psaL**	**TFIGNLPAYR**	**70**
		**TFIGNPAYRK**	**23**
		**AESNQVVQAYNGDPFVGHLSTPISDSAFTR**	**5**

sll0851	CP43	NDIQPWQVR	57
		GPNGLDLDKLR	52
		LGANIASAQGPTGLGK	94
		DLPSTGFAWWSGNAR	87
		GIDRETEPTLFMPDLD	32
		VITNPTLNPAIIFGYLLK	58
slr0906	CP47	GYFQEEIQR	71
		VHTVVLNDPGR	18
		GYFQEEIQRR	24
		LAFYDYVGNSPAK	92
		YQWDKGYFQEEIQR	101
		VDSQLAEGASLSEAWSTIPEK	31
		DVFAGVDPGLEEQVEFGVFAK	122
		AQLGEGFDFDTETFNSDGVFR	102
		RVDSQLAEGASLSEAWSTIPEK	78
		KAQLGEGFDFDTETFNSDGVFR	149
		FSVEQTGVTVSFYGGALDGQTFSNPSDVKK	22

ssl2598	psbH	**LGDILRPLNSEYGK**	**65**
ssr3451	psbE	**QELPILQER**	**57**
sll0849	psbD	**AYDFVSQELR**	**23**
		**AAEDPEFETFYTK**	**67**
slr1311/sll1867	psbA2/ psbA3	**VIGTWADVLNR**	**62**
		**LIFQYASFNNSR**	**61**
slr1326	atpA	**WDSLGRPIDGK**	**22**
		**LLESPAPGIIER**	**51**
slr1329	atpB	**FVQAGSEVSALLGR**	**61**
		**DVNKQDVLLFIDNIFR**	**47**
		**MPSAVGYQPTLGTDVGDLQER**	**20**
		**NSAGNEVAVTCEVQQLLGDNQVR**	**34**
slr1327	atpC	**LSFAETELPLFEQRFVSLISSQPVVQ**	**48**
		**TLFPLSPQGLEAPDDEIFR**	**13**
slr0228	FtsH2	**SRVDLPTNAPELIAR**	**22**
		**LVDLLIEKETIDGEEFR**	**41**

		**TPGFSGADLANLLNEAAILTAR**	**7**
sll1463	**FtsH4**	**AAEEIVFDSITTGAANDLQR**	**70**
		**TPGFAGADLANLVNEAALLEER**	**25**
sll0247	**isiA**	**RVEQAFDSLQT**	**83**
sll1578	**cpcA**	**FLSSTELQIAFGR**	**103**
slr1128	**hypothetical**	**DSAINSAQGDAQAR**	**18**
